# Teaching Anorectal Malformations

**Published:** 2015-07-01

**Authors:** G Raghavendra Prasad

**Affiliations:** Department of Pediatric Surgery, Deccan College of Medical Sciences and Princess Esra Hospital, Hyderabad, India

**Dear Sir**

I was following Dr. Sushmita Bhatnagar's tutorials. [1,2] It is conventional, although necessary to teach as a primary need. I feel teaching anorectal malformations should include write up on issues of continence and constipations as these are lifelong problems in these patients. Teaching anatomy and physiology of continence and various techniques to manage incontinence or constipations is of prudent importance. Similarly role of CT/MRI, laparoscopic procedures, bowel training program, parental counseling etc. are important aspects in the management of these patients and should also be included in Face the Examiner Section.

## Footnotes

**Source of Support:** Nil

**Conflict of Interest:** None

